# Thoracoscopic Resection of a Rare Case of Hemangioma of the Azygos Venous Arch

**DOI:** 10.1155/2017/1693912

**Published:** 2017-03-07

**Authors:** Ma Husai, Cai Yixin, Zhang Ni, Wang Wenxin, Fu Xiangning

**Affiliations:** ^1^Department of Thoracic Surgery, Qinghai People's Hospital, No. 2 Gonghe Rd, Xining, Qinghai 810001, China; ^2^Department of Thoracic Surgery, Tongji Hospital, Tongji Medical College, Huazhong University of Science and Technology, Wuhan, Hubei 430030, China; ^3^Department of Thoracic Surgery, Qinghai Red Cross Hospital, No. 55 South Street, Xining, Qinghai 810000, China

## Abstract

Hemangioma of the azygos venous arch is an exceedingly rare incident. This is a case of a thoracoscopic complete resection of a hemangioma of the azygos venous arch in a 37-year-old woman.

## 1. Introduction

Hemangioma of the azygos venous arch may be caused by venous malformation, tumor, or local thrombosis [1, 2]. It is extremely rare and there are very few documented cases described in medical literature [1]. Surgery is required for successful treatment of this disease. This case was reported in order to describe and discuss the strategy that was used for successful diagnosis and therapy.

## 2. Case Report

**Figure 1:**
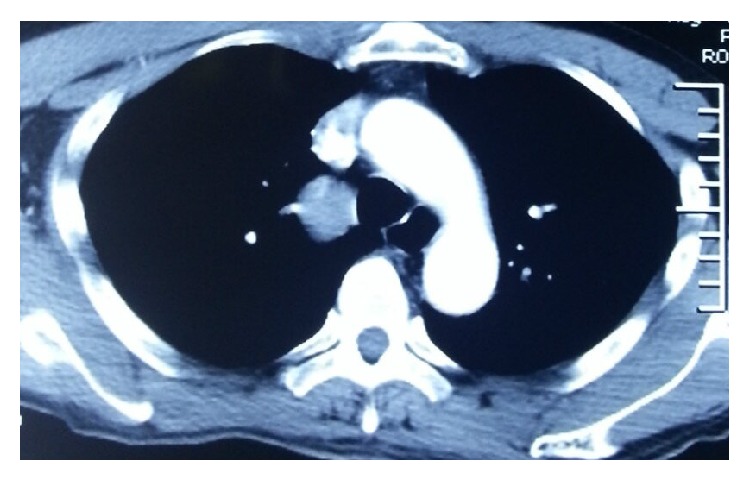
Contrast computed tomography confirmed a slowly enhancing mass with a smooth margin adjacent to the right side of the trachea in the pathway of the azygos arch.

**Figure 2:**
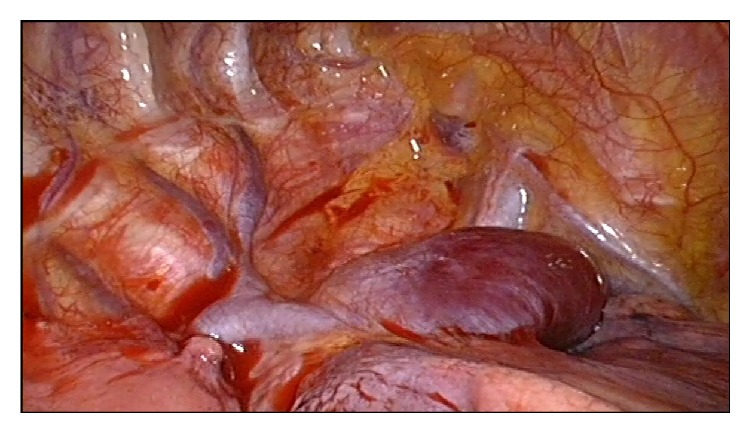
Thoracoscopic surgery revealed a mass arising from the azygos venous arch; there was light adhesion.

**Figure 3:**
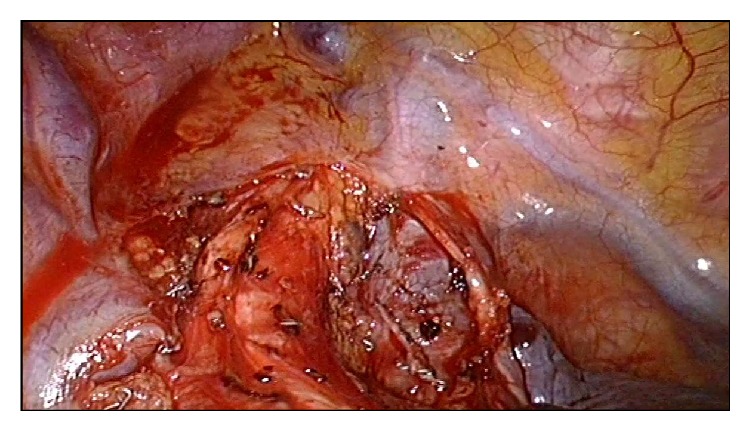
A complete resection of the venous mass was performed.

**Figure 4:**
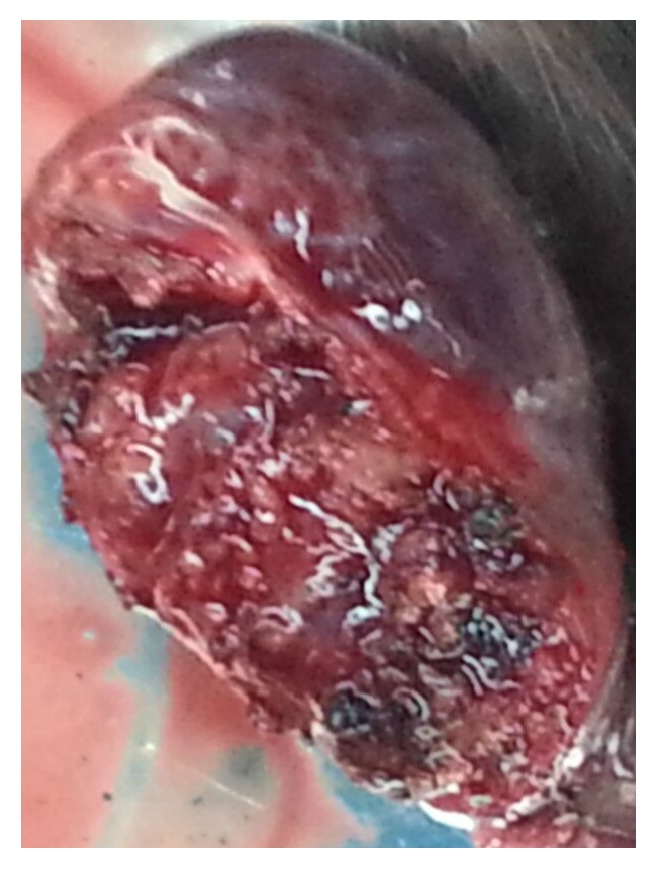
A complete resection of 4.2 cm mass of the azygos venous arch was performed and some thrombosis within the mass was observed.

A 37-year-old woman arrived at our hospital for a routine physical examination and chest X-ray. She had no diseases previously; she had never smoked and never been exposed to noxious agents. Her physical examination and hematologic test results were normal. The chest X-ray reading showed a slightly widening superior mediastinum with a possible mass. A noncontrast chest computed tomography (CT) scan showed a right posterior mediastinal mass with a smooth margin adjacent to the trachea. A contrast CT scan confirmed a slowly enhancing mass in the pathway of the azygos venous arch (Figure 1). Magnetic resonance imaging (MRI) showed mixed signals and a partially low void within the mass. These findings indicated that this was a vascular mass originating from the azygos venous arch. The patient was admitted for VATS triportal surgery. We use two universal staplers of Endo GIA Ultra to resect the vascular tumor. Thoracoscopic surgery revealed a mass arising from the azygos venous arch; there was light adhesion (Figure 2). A complete resection of 4.2 cm mass of the azygos venous arch was performed (Figure 3) and some thrombosis within the mass was observed (Figure 4). Pathological examination confirmed the diagnosis of a hemangioma. The patient had an uneventful postoperative recovery and was discharged on the seventh day after surgery. The last follow-up 12 months after the surgery revealed the patient was well without any discomfort.

## 3. Discussion

Hemangioma of the azygos venous arch is an extremely rare incident [1]. Patients with hemangioma are often asymptomatic and detection is often an incidental finding obtained during a general examination or during another medical procedure. Sometimes there are clinical manifestations due to the tumor compressing surrounding organs [3]. Diagnosis of this disease is aided by the use of chest X-ray, CT, MRI, transesophageal echography, endobronchial ultrasonography, and angiography [4, 5]. However, it is difficult to diagnose this disease with imaging alone. A definite diagnosis is often made by exploratory surgery and pathological diagnosis. As hemangioma of the azygos venous arch is very rare, differential diagnosis would include pericardial cyst, tracheal cyst, Castleman's disease, and neurogenic tumor if the tumor presents adjacent to the trachea and posterior mediastinal mass [6].

Hemangioma of the azygos venous arch should be surgically resected due to the potential of compression symptoms, bleeding, or embolization [7]. The mass of our patient was completely resected through thoracoscopic surgery. It should be kept in mind that for this surgical procedure preparations should be made for a conversion to thoracotomy in order to prevent possible massive intraoperative hemorrhage. Because the hemangioma wall is delicately thin, it is very easy to inadvertently cause unwanted bleeding. Therefore, unnecessary intraoperative manipulation (either pinching or pressure) of the mass should be avoided to prevent hemorrhagic complications. Consideration should be made to deal with the proximal hemangioma first to prevent thrombosis from the hemangioma entering into the superior vena cava and right atrium. The hemangioma should be carefully dissected to avoid rupture. Otherwise this would result in a blood obscured surgical field which would increase the potential for harm to vital organs and vessels. This is especially relevant when there is adhesion to the superior vena cava and right pulmonary artery. The superior vena cava should be protected during surgery in order to avoid postoperative stenosis.
